# Rehabilitation and Physiotherapy for Functional Sequelae in Adults with Head and Neck Cancer: A Systematic Review and Meta-Analysis

**DOI:** 10.3390/medicina62091648

**Published:** 2026-08-28

**Authors:** Miriam Linero-Bocanegra, Carlos Fernández-Morales, Daniel Torres-Lagares, Elena Oliva-Ferrusola, Cristo Jesús Barrios-Quinta, Ángel Rufino Yáñez-Álvarez

**Affiliations:** 1 Department of Physiotherapy, University of Seville, 41009 Seville, Spain; mlinerobocanegra@gmail.com (M.L.-B.); cristo.barrios@gmail.com (C.J.B.-Q.); ayalvarez@us.es (Á.R.Y.-Á.); 2 Department of Medical-Surgical Therapy, Faculty of Medicine and Health Sciences, University of Extremadura, 06006 Badajoz, Spain; 3 Department of Dentistry, Faculty of Dentistry, University of Seville, 41009 Seville, Spain; danieltl@us.es (D.T.-L.); elenaof2209@gmail.com (E.O.-F.)

**Keywords:** head and neck cancer, rehabilitation, physiotherapy

## Abstract

*Background and Objectives:* Functional sequelae following head and neck cancer (HNC) treatment frequently impair swallowing, mouth opening, speech, cervical and shoulder mobility, and overall quality of life. Although rehabilitation is increasingly recognized as an essential component of multidisciplinary care, evidence has largely focused on isolated functional impairments rather than providing a comprehensive evaluation across rehabilitation domains. To systematically evaluate the effectiveness of rehabilitation and physiotherapy interventions for preventing and treating functional sequelae in adults with head and neck cancer. *Materials and Methods:* A systematic review and meta-analysis of randomized controlled trials was conducted according to PRISMA 2020 guidelines. PubMed/MEDLINE, Embase, Cochrane Library, and PEDro were searched from inception to June 2026. Studies involving adult patients with head and neck cancer receiving conservative rehabilitation interventions were included. Random-effects meta-analyses were performed for clinically homogeneous intervention contrasts within the mouth-opening and trismus domains. Distinct comparisons involving intervention timing, additional therapeutic components, or delivery strategies were analysed separately. *Results:* Twenty-seven publications representing 21 independent randomized trials were included. Quantitative synthesis was restricted to clinically homogeneous intervention contrasts. Compared with usual care, preventive jaw rehabilitation favoured greater mouth opening across three independent trials (mean difference = 2.85 mm; conventional 95% CI: 0.38–5.32), although the modified Hartung–Knapp sensitivity interval included no effect (95% CI: −2.58–8.27). The pooled estimate for trismus across three independent trials also favoured rehabilitation, although both conventional and small-sample-adjusted confidence intervals included no effect (RR = 0.87; conventional 95% CI: 0.53–1.42; modified Hartung–Knapp 95% CI: 0.25–2.98). Postoperative swallowing rehabilitation after oral-cancer surgery and free-flap reconstruction favoured rehabilitation in both sequential cohorts (adjusted difference in change = 10.06 points; 95% CI: 7.68–12.44; GEE group-by-time β = 7.29 points; 95% CI: 4.11–10.47). Additional timing, support, enhancement, and delivery comparisons were analysed separately. *Conclusions:* Targeted rehabilitation interventions produced favourable estimates for mandibular mobility and postoperative swallowing function in adults with head and neck cancer. Preventive jaw rehabilitation favoured greater mouth opening, although the result was inconclusive after small-sample adjustment; the trismus estimate also favoured rehabilitation but remained imprecise.

## 1. Introduction

Head and neck cancer (HNC) represents one of the major global public health challenges, accounting for more than 900,000 new cases and approximately 450,000 deaths annually [1]. This heterogeneous group of malignancies includes tumors arising from the oral cavity, oropharynx, hypopharynx, larynx, nasopharynx, nasal cavity, and paranasal sinuses. Although tobacco and alcohol consumption remains the leading etiological factor, the incidence of human papillomavirus (HPV)-related tumors, particularly oropharyngeal cancer, has increased substantially over recent decades [2].

Advances in early diagnosis, reconstructive surgical techniques, intensity-modulated radiotherapy, systemic therapies, and multidisciplinary care have significantly improved survival among patients with head and neck cancer [2]. Consequently, the number of survivors continues to increase, and clinical attention has shifted from focusing exclusively on tumor control to addressing functional sequelae and health-related quality of life following treatment [3,4].

However, treatment for head and neck cancer frequently results in complex anatomical and functional impairments arising from both the disease itself and its treatment, including surgery, radiotherapy, and systemic therapies [5,6]. The most common sequelae include trismus, dysphagia, restricted cervical and shoulder mobility, lymphedema, musculoskeletal pain, voice and speech disorders, fatigue, and reduced health-related quality of life [5,7]. These complications may persist for months or even years after completion of treatment, compromising essential functions such as eating, communication, oral hygiene, breathing, and social participation [8,9].

In this context, rehabilitation has assumed an increasingly important role in the comprehensive management of head and neck cancer [3,8]. Conservative rehabilitation strategies include therapeutic exercise, mandibular rehabilitation, manual therapy, swallowing exercises, cervical and shoulder rehabilitation, supervised home-based exercise programmes, telerehabilitation, and interventions aimed at preventing or minimizing treatment-related functional sequelae [6,10]. Nevertheless, considerable variability exists among published rehabilitation programmes with regard to the timing of intervention, treatment intensity, duration, therapeutic components, and outcome measures [6,11].

During the past decade, the number of clinical trials and systematic reviews investigating rehabilitation in patients with head and neck cancer has increased substantially [11]. Nevertheless, most published reviews have focused on specific sequelae, particularly treatment-induced trismus or dysphagia rehabilitation, whereas others have been restricted to specific patient populations or isolated rehabilitation interventions [11]. Although these studies have provided valuable insights, they offer only a partial view of rehabilitation and limit a comprehensive understanding of the currently available evidence [8].

Although systematic reviews addressing specific functional sequelae are available, to the best of our knowledge, no systematic review and meta-analysis has comprehensively synthesized the evidence from randomized controlled trials evaluating rehabilitation interventions across the full spectrum of functional sequelae associated with head and neck cancer. Such an approach integrates both preventive and therapeutic rehabilitation strategies while considering the different functional domains affected by the disease and its treatment.

Therefore, the aim of the present systematic review and meta-analysis was to synthesize and quantitatively evaluate the evidence from randomized controlled trials regarding the effectiveness of rehabilitation and physiotherapy interventions for functional sequelae associated with head and neck cancer in adult patients. As a secondary objective, differences according to the functional domain assessed, the type of rehabilitation intervention, and the clinical context in which rehabilitation was delivered were explored.

## 2. Materials and Methods

### 2.1. Study Design

This systematic review and meta-analysis was conducted in accordance with the Preferred Reporting Items for Systematic Reviews and Meta-Analyses (PRISMA 2020) statement [12] and the methodological recommendations of the Cochrane Handbook for Systematic Reviews of Interventions (Version 6.5) [13]. The review protocol was prospectively registered in the International Prospective Register of Systematic Reviews (PROSPERO; CRD420261425384) [14].

The research question was formulated according to the PICO framework (Population, Intervention, Comparator, and Outcomes).

### 2.2. Search Strategy

A systematic literature search was conducted in PubMed/MEDLINE, Embase, Cochrane Library, and PEDro from database inception to 10 June 2026.

The search strategy combined controlled vocabulary terms (MeSH and Emtree) and free-text keywords related to head and neck cancer (e.g., “Head and Neck Neoplasms”, “head and neck cancer”, “oral cancer”, “oropharyngeal cancer”, and “laryngeal cancer”) with terms describing rehabilitation interventions (e.g., “Rehabilitation”, “Physical Therapy Modalities”, “physiotherapy”, “exercise therapy”, “manual therapy”, “telerehabilitation”, and “home exercise”). Boolean operators (AND and OR) were adapted to the indexing system of each database.

After combining both search concepts, validated randomized-trial filters were applied in PubMed/MEDLINE and Embase; CENTRAL was searched without an additional trial filter, and PEDro results were restricted using its clinical-trial method filter to identify randomized controlled trials. In addition, the reference lists of the included studies and relevant systematic reviews were manually screened to identify potentially eligible publications that had not been retrieved through the electronic database search.

The complete search strategy for each database is provided in Supplementary Material S1.

### 2.3. Eligibility Criteria

Eligibility criteria were established according to the PICO framework (Population, Intervention, Comparator, and Outcomes).

#### 2.3.1. Population

Studies involving adult patients (≥18 years) with a diagnosis of primary head and neck cancer who had undergone surgery, radiotherapy, chemotherapy, chemoradiotherapy, immunotherapy, or any combination of oncological treatments were eligible. Included studies were required to evaluate at least one functional, musculoskeletal, or participation-related sequela resulting from the disease or its treatment.

#### 2.3.2. Intervention

Conservative rehabilitation or physiotherapy interventions aimed at preventing or treating functional sequelae associated with head and neck cancer were eligible. These interventions included, but were not limited to, therapeutic exercise, manual therapy, electrotherapy, functional training, orofacial rehabilitation, cervical and shoulder rehabilitation, swallowing exercises, supervised home-based exercise programmes, and telerehabilitation. Studies evaluating specific rehabilitation devices (e.g., jaw-mobilization devices or exercise-assistance devices) were also included, provided that these devices formed part of a conservative rehabilitation programme targeting functional recovery.

Eligible comparator groups included usual care, standard education or instructions, no intervention, waiting-list controls, placebo or sham interventions, other active interventions, and different rehabilitation modalities, treatment intensities, frequencies, or dosages.

#### 2.3.3. Outcomes

Studies were required to report at least one validated functional outcome related to head and neck cancer or its treatment. Outcomes of interest included, but were not limited to, mouth opening, mandibular function, swallowing function, cervical mobility, shoulder function, voice, fatigue, lymphedema, and health-related quality of life.

#### 2.3.4. Exclusion Criteria

• Studies were excluded if they:
• Included participants without a diagnosis of head and neck cancer.
• Evaluated temporomandibular disorders, bruxism, myofascial pain, orthognathic surgery, mandibular trauma, or oral fibrosis unrelated to oncological treatment.
• Did not assess functional sequelae attributable to head and neck cancer or its treatment.
• Evaluated general rehabilitation interventions without reporting outcomes specifically related to functional sequelae associated with head and neck cancer.
• Assessed exclusively surgical, pharmacological, dental, or prosthetic interventions without a rehabilitation component aimed at functional recovery.
• Did not include a comparator group.
• Were observational studies, cross-sectional studies, case series, case reports, reviews, study protocols, editorials, letters to the editor, expert opinions, or conference abstracts without complete data.
• Did not provide extractable data for at least one eligible functional outcome.
• Were conducted in animal models or preclinical settings.
• Were published in languages other than English or Spanish.

### 2.4. Study Selection

The search results were initially exported to reference management software to identify and remove duplicate records. Subsequently, the references were imported into Rayyan (web version; Rayyan Systems Inc., Cambridge, MA, USA), an online platform used to facilitate the screening process and identify any remaining duplicate records [15].

Study selection was conducted in two stages:

• Screening of titles and abstracts.
• Full-text assessment for eligibility.

Both stages were performed independently by two reviewers using the predefined eligibility criteria. Any disagreements were resolved through discussion and consensus. When consensus could not be reached, a third reviewer was consulted.

**Figure 1:**
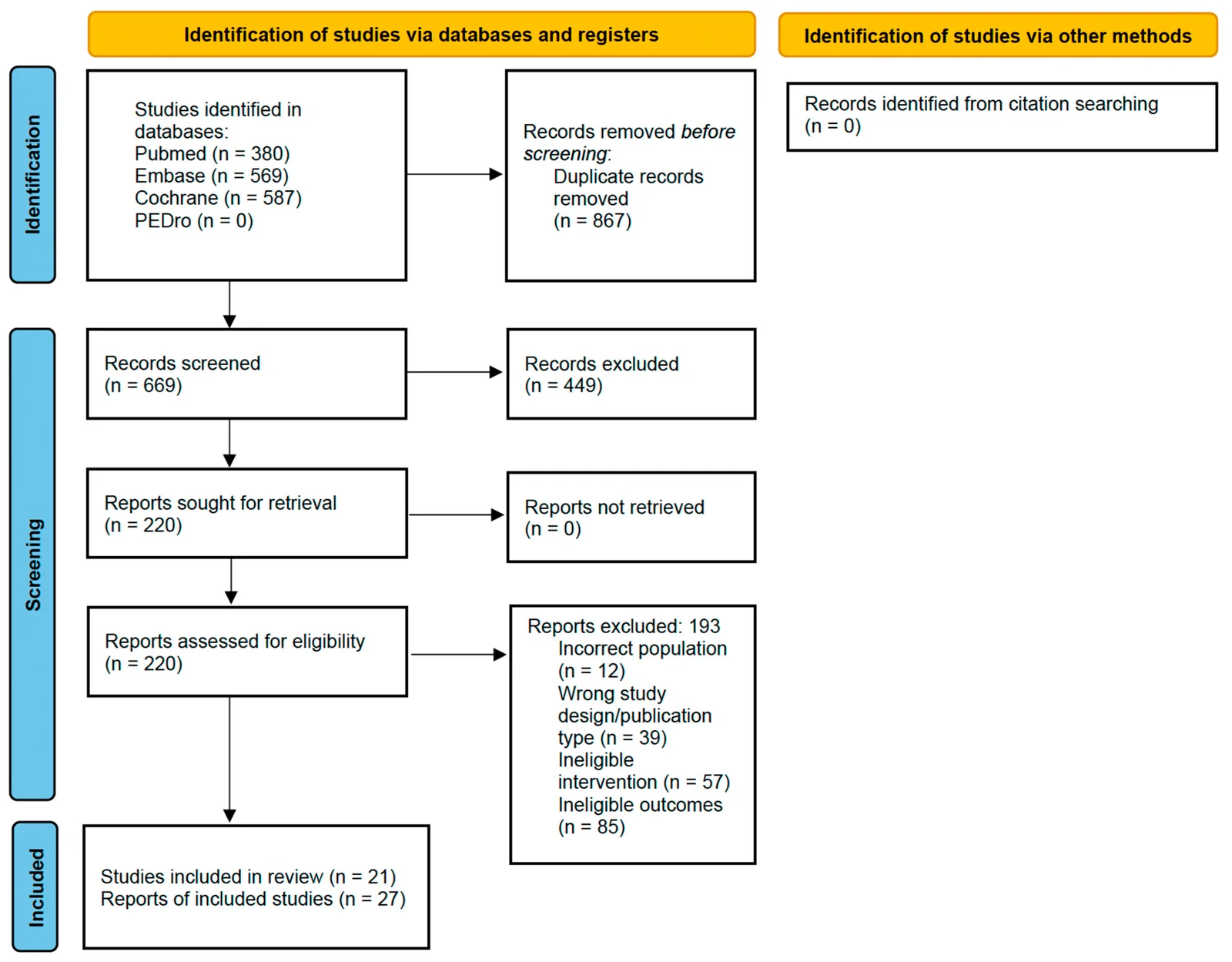
PRISMA 2020 flow diagram of the study selection process.

The complete study selection process was documented using a PRISMA 2020 flow diagram (Figure 1).

### 2.5. Data Extraction

Data were extracted using a standardized data extraction form specifically developed and pilot-tested for this systematic review.

Data extraction was performed by one reviewer and independently verified by a second reviewer.

The following information was collected from each included study:

• First author and year of publication, unique trial identifier, and report type (primary report, companion publication, secondary analysis, or long-term follow-up report);
• Country;
• Study design;
• Sample characteristics;
• Tumor site;
• Oncological treatment received;
• Characteristics of the rehabilitation intervention;
• Characteristics of the comparator group;
• Duration of follow-up;
• Outcome measures and assessment instruments;
• Quantitative results for the outcomes of interest;
• Data required for the meta-analysis (means, standard deviations, sample sizes, and event counts).

Companion publications, secondary analyses, and long-term follow-up reports arising from the same randomized population were linked under a common trial entry and treated as a single independent trial. When several reports provided data for the same comparison, outcome, and follow-up period, participants were included only once in each quantitative synthesis.

### 2.6. Risk of Bias Assessment

Risk of bias was assessed for each specific result included in the meta-analyses and for separately reported quantitative estimates using the revised Cochrane risk-of-bias tool for randomized trials (RoB 2) [16].

Each assessment was defined according to the randomized comparison, outcome, follow-up time, and effect of interest. The effect of assignment to intervention was assessed in all cases.

The standard RoB 2 tool was used for individually randomized parallel-group trials. The cluster-randomized version of RoB 2 was used for cluster-randomized trials [17] and additionally considered bias arising from the identification or recruitment of participants within clusters. Domain-level judgements were classified as low risk, some concerns, or high risk of bias, and an overall judgement was assigned to each result according to the RoB 2 algorithms.

Risk of bias was assessed independently by two reviewers (M.L.-B. and C.F.-M.). Disagreements were resolved through discussion and consensus; when consensus could not be reached, a third reviewer (Á.R.Y.-Á.) was consulted. Detailed domain-level judgements and the reasons supporting each assessment are provided in Supplementary Table S2. Risk-of-bias figures were generated using robvis version 0.3.1 [18].

### 2.7. Data Synthesis and Statistical Analysis

Quantitative syntheses were performed when at least two independent trials reported comparable outcomes for the same randomized intervention contrast, functional domain, clinical context, and follow-up period. The PROSPERO registration prespecified the functional outcome domains and quantitative synthesis of sufficiently comparable studies. The specific outcome-selection hierarchy, common follow-up rules, and organization by randomized intervention contrast were developed as operational refinements during the review process and were not prespecified in the registration. Each independent trial contributed a maximum of one effect estimate to each meta-analysis. Companion publications, secondary analyses, and follow-up reports were linked under a common trial entry to prevent duplication of participants. Meta-analyses were conducted in R version 4.5.1 (R Foundation for Statistical Computing, Vienna, Austria) using the metafor package version 4.8-0 [19]. Clinical comparability was determined by considering the tumour site and oncological treatment pathway, the phase of care in which rehabilitation was delivered, the functional impairment addressed, and the follow-up period. Interventions delivered as prehabilitation, during oncological treatment, in the postoperative period, or during long-term survivorship were not pooled solely because they assessed the same broad functional domain.

Intervention contrasts were classified as rehabilitation versus no rehabilitation or usual care, early versus delayed rehabilitation, basic versus enhanced rehabilitation, the same programme with versus without additional support, and one active rehabilitation modality or delivery strategy versus another. Trials belonging to different contrast categories were analysed separately. When fewer than two clinically comparable trials were available, effect estimates were presented individually or the findings were synthesized narratively.

Outcome selection followed a predefined hierarchy. Measures directly assessing the functional domain of interest were prioritized, followed by the primary outcome identified by the trial. When several eligible measures remained, objective or clinician-assessed measures were selected before patient-reported outcomes, and function-specific measures were selected before general quality-of-life measures. Direct measurements in millimetres were selected for mouth opening, dichotomous definitions based on a maximum mouth opening of 35 mm or less were selected for trismus, and the MASA-OC total score was selected for postoperative swallowing function.

Trials were pooled only when they reported outcomes within the same clinically relevant follow-up period. The longest common follow-up was selected when several comparable assessments were available. The mouth-opening and trismus meta-analyses used outcomes assessed 12 months after radiotherapy, whereas the postoperative swallowing estimates were reported at the 1-month assessment after surgery. Outcomes from other follow-up periods were analysed separately or synthesized narratively.

Changes from baseline were prioritized for continuous outcomes when an estimate and its variance were available. Endpoint values were used when change scores were unavailable, provided that the studies reported the outcome in the same measurement unit. An endpoint-only sensitivity analysis was performed for mouth opening. Outcome-specific sample sizes were based on the analytic population reported for each outcome and follow-up in the original trial.

Continuous outcomes reported on the same scale were pooled as mean differences. Dichotomous outcomes were pooled as risk ratios. Positive mean differences favoured rehabilitation, whereas risk ratios below 1 favoured rehabilitation for adverse functional outcomes. Random-effects models were fitted using restricted maximum-likelihood estimation. For the primary analyses, confidence intervals and *p* values for the pooled effects were calculated using Wald-type inference based on the model standard error and standard normal quantiles. Because each meta-analysis included only three trials, robustness to the inferential method was examined using a modified Hartung–Knapp adjustment, with the same REML estimate of τ^2^, the variance scale factor constrained to be at least 1, and a t distribution with k − 1 degrees of freedom [13,19]. This adjustment was treated as a sensitivity analysis because it may be highly conservative with very small k. Statistical heterogeneity was assessed using Cochran’s Q test, I^2^, and τ^2^. Prediction intervals were calculated using the conventional plug-in normal approximation based on the REML estimate of τ^2^ when at least three independent trials were available [13]. Because only three trials contributed to each meta-analysis, prediction intervals were considered descriptive and interpreted with explicit caution. In multi-arm trials, clinically comparable intervention groups were combined for the principal analysis so that a shared control group was included only once. In the three-arm trial by Bragante et al., the two active intervention groups were combined for the principal mouth-opening and trismus analyses. Each active arm was subsequently compared separately with the control group in sensitivity analyses.

Cluster-randomized postoperative swallowing estimates were reported separately using the model-based coefficients provided by the original investigators. For Zhang et al. [20], the extracted coefficient was the between-group difference in MASA-OC change from postoperative day 6 to day 30 (10.06 points, 95% CI 7.68–12.44) from a linear mixed-effects model with room number as a random effect and the day-6 MASA-OC score as a covariate; the report did not provide further details on the within-participant covariance structure. For Wu et al. [21], the extracted coefficient was the GEE group-by-time interaction for intervention ×1 month (β = 7.289, SE = 1.621), with the control group and postoperative day 6 as reference categories. GEE was used for repeated MASA-OC measurements, but the report did not specify additional covariates, the working correlation structure, or whether ward-room clustering was incorporated. Because the variance estimates could not be established as sufficiently comparable, the two estimates were not pooled. Publication bias was not formally assessed because fewer than ten independent trials contributed to each meta-analysis [13].

## 3. Results

### 3.1. Study Selection

The database search identified 1536 records. After removal of 867 duplicate records, 669 records were screened by title and abstract. Of these, 449 records were excluded, and 220 full-text reports were assessed for eligibility. A total of 193 reports were excluded for the following reasons: incorrect population (n = 12), wrong study design or publication type (n = 39), ineligible intervention (n = 57), and ineligible outcomes (n = 85). Twenty-seven publications representing 21 independent randomized trials were included in the systematic review. Fourteen trials provided extractable quantitative estimates, including three trials that contributed to the two clinically homogeneous meta-analyses. The study selection process is presented in Figure 1.

### 3.2. Characteristics of the Included Studies

The 21 independent randomized trials evaluated rehabilitation across a broad range of functional domains in patients with head and neck cancer. These included mouth opening and trismus prevention, swallowing rehabilitation during or after oncological treatment, postoperative rehabilitation after oral-cancer surgery, prehabilitation, voice and communication rehabilitation, and interventions designed to improve treatment delivery or adherence. This broad clinical scope was retained at the systematic-review level, whereas quantitative synthesis was restricted to trials addressing the same rehabilitation question within a comparable oncological treatment pathway and phase of care (Table 1).

Jaw-related trials evaluated preventive jaw rehabilitation versus usual care, early versus delayed initiation of the same programme, structured telephone support added to a mouth-opening programme, and comparisons between active jaw-mobilization modalities [22,23,24,25,26,27,28].

Swallowing trials covered preventive exercise during radiotherapy or chemoradiotherapy, prehabilitation, rehabilitation of established dysphagia, postoperative rehabilitation after oral-cancer surgery and free-flap reconstruction, and comparisons between basic and enhanced programmes or alternative delivery strategies [20,21,29,30,31,32,33,34,35,36,37,38,39,40].

Voice and communication outcomes were reported after radiotherapy for laryngeal cancer and after total laryngectomy [41,42,43,44,45,46]. Related reports arising from the same randomized population were grouped under a common trial entry. These studies were synthesized narratively because the interventions, clinical contexts, and endpoints were not sufficiently comparable for meta-analysis.

**Table 1:** Characteristics of the independent randomized trials and associated publications.

Trial and Associated Publication(s)	Country	Design	Population/Treatment Context	Sample Size	Intervention	Comparator	Main Domain	Follow-Up/Main Timepoints	Synthesis in Review
Yang et al., 2025 [22]	China	Randomized controlled trial; blinded outcome assessors/statistical analysts	Oral squamous cell carcinoma after surgery and postoperative IMRT	76 randomized; 36/36 analysed	Mouth-opening protocol started in postoperative week 2	Same protocol started in week 4	Mouth opening/trismus	Up to 12 weeks after surgery	Separate quantitative estimate: early versus delayed rehabilitation
Schipor-Diaconu et al., 2024 [29]	Romania	Randomized controlled trial	Stage III-IV laryngeal cancer undergoing total laryngectomy	96 randomized; 46/46 analysed	Prophylactic swallowing exercises after surgery	No preventive exercises; treatment as needed	Swallowing after laryngectomy	3, 6, 9, and 12 months	Narrative synthesis
Høgdal et al., 2015 [23]	Denmark	Parallel-group randomized clinical trial; blinded outcome assessment	Oral cavity or oropharyngeal cancer treated with curative RT ± chemotherapy/surgery	100 randomized; 36/34 in complete-case primary analysis	Preventive physiotherapist-supervised exercises during RT plus home programme	Usual care	Mouth opening/trismus prevention	5 and 12 months after RT	Meta-analysis: mouth opening and trismus
Swedish voice rehabilitation trial (Tuomi et al., 2014; Karlsson et al., 2015; Tuomi et al., 2017; Millgård et al., 2020; Karlsson et al., 2024) [41,42,43,44,45]	Sweden	Randomized voice-rehabilitation trial with companion, subgroup, and long-term follow-up reports	Laryngeal cancer treated with curative RT ± chemotherapy	89 randomized; 37/37 in main trial analyses; smaller subsets at later follow-up	Ten-session structured voice rehabilitation after RT	No structured voice rehabilitation; vocal-hygiene advice	Voice/communication/HRQL	1, 6, 12, 24, and 60 months after RT	Narrative synthesis; trial counted once
Li et al., 2025 [46]	China	Randomized controlled trial	Patients after total laryngectomy	70 randomized; 35/35 analysed	Esophageal speech rehabilitation programme plus conventional care	Conventional nursing care	Esophageal speech/voice/quality of life	1, 6, and 12 months	Narrative synthesis
Bragante et al., 2020 [24]	Brazil	Three-arm randomized controlled trial	Head and neck cancer undergoing definitive or postoperative external-beam RT	90 randomized; 30/30/30	Jaw exercises with hyperboloid device, with or without TheraBite	Usual-care guidance	Mouth opening/trismus prevention	End of RT and 12 months after RT	Meta-analysis: mouth opening and trismus; active arms combined
Carnaby-Mann et al., 2012 [30]	USA	Three-arm randomized phase II trial	Predominantly oropharyngeal HNC undergoing RT ± chemotherapy	58 randomized; 14 active/14 usual care/13 sham assessed at 6 weeks	High-intensity swallowing exercise programme (Pharyngocise)	Usual care; separate sham-intervention arm	Swallowing; secondary mouth opening	End of CRT/6 weeks and 6 months	Separate quantitative estimates
Lazarus et al., 2014 [31]	USA	Two-arm randomized clinical trial; blinded swallowing assessment	Stage II-IV oral or oropharyngeal cancer treated with RT ± chemotherapy	23 randomized; 8/10 analysed	Traditional exercises plus isometric tongue-resistance exercises	Traditional tongue/laryngeal exercises	Swallowing/tongue strength	10 weeks after RT/CRT	Separate quantitative estimate: enhanced versus basic rehabilitation
Zhang free-flap rehabilitation trial (Zhang et al., 2022; Zhang et al., 2024) [20,32]	China	Cluster-randomized controlled trial; ward rooms as the unit of randomization (18 clusters); companion outcome reports	Oral cancer after extended resection and free-flap reconstruction; single centre, Peking University School and Hospital of Stomatology; recruited August 2021–January 2022	123 screened; 75 randomized (37/38); 68 completed the 1-month assessment (34/34)	Personalized swallowing/oral exercises from postoperative day 6	Routine nursing and swallowing-related education	Postoperative swallowing/oral function/quality of life	Postoperative days 15 and 30/1 month	Separate quantitative estimate: postoperative MASA-OC; companion reports counted once
Loorents et al., 2014 [25]	Sweden	Multicentre randomized study	HNC receiving RT with dose to jaw muscles/TMJ and baseline MIO > 35 mm	66 randomized; 23/19 at 12 months	Prophylactic TheraBite training during and after RT	Standard treatment without prophylactic TheraBite	Mouth opening/trismus prevention	Weekly during RT; 3, 6, and 12 months after RT	Narrative synthesis
Langmore multicentre NMES trial (Krisciunas et al., 2017, secondary analysis) [33]	USA	Secondary compliance analysis of a multicentre randomized trial	HNC survivors with long-term radiation-associated dysphagia	170 randomized in parent trial; 153 included in compliance analysis	Swallowing exercises plus active NMES	Same exercises plus sham NMES	Post-treatment dysphagia/adjunct NMES/adherence	Week 13 after a 12-week programme	Narrative synthesis: active NMES versus sham NMES
Messing et al., 2017 [34]	USA	Randomized controlled trial; intention-to-treat analysis	Stage III-IV HNSCC undergoing curative chemoradiotherapy	60 randomized; 29/28 analysed	Prophylactic swallow therapy plus TheraBite and weekly SLP support	No direct prophylactic SLP therapy; minimum TheraBite standard	Swallowing; secondary mouth opening	3, 6, 12, and 24 months after CRT	Separate quantitative estimate: enhanced versus basic rehabilitation
Baudelet et al., 2024 (PRESTO trial) [35]	Belgium	Multicentre three-arm randomized controlled trial	Stage III or IVA-B oropharyngeal cancer treated with RT/CRT	148 randomized; 49/49/50	Same prophylactic swallowing programme delivered by paper, app, or therapist	Alternative delivery modes of the same active programme	Swallowing prophylaxis/adherence/delivery mode	End of RT/CRT; 1 and 3 months after treatment	Narrative synthesis: delivery-strategy comparison
van der Geer et al., 2020 [26]	Netherlands	Randomized controlled trial; active-device comparison	HNC survivors with established trismus (MMO ≤ 35 mm)	27 enrolled; 22 started treatment; 12 completed follow-up	TheraBite stretching exercises	Dynasplint Trismus System	Treatment of established trismus	Three-month exercise protocol	Narrative synthesis: active-device comparison
Govender et al., 2020 [36]	United Kingdom	Two-arm randomized feasibility trial	Newly diagnosed stage III/IV HNC before oncological treatment	32 randomized; 16/16 analysed	Tailored SIP SMART pre-treatment swallowing intervention	Usual care and generic advice/exercises when relevant	Swallowing prehabilitation	1, 3, and 6 months after treatment	Separate quantitative estimate: prehabilitation versus usual care
Swedish head-lift exercise trial (Tuomi et al., 2022; Petersson et al., 2023) [37,38]	Sweden	Randomized controlled trial with companion long-term HRQL report	HNC survivors with radiation-induced dysphagia 6–36 months after RT/CRT	61 randomized; 25/27 at 8 weeks and 19/24 at 12 months	Shaker head-lift exercise plus standard dysphagia care	Standard dysphagia care	Post-treatment dysphagia/swallowing-related HRQL	8 weeks and 12 months	Separate quantitative estimate: enhanced versus basic rehabilitation; trial counted once
Zhang Y et al., 2025 [39]	China	Prospective randomized parallel-controlled trial	Primary HNC undergoing particle therapy ± chemotherapy	95 analysed; 47/48	Feeding management plus swallowing training during particle therapy	Routine radiotherapy care and health education	Swallowing during particle therapy	Post-treatment and 3 months	Separate quantitative estimate: rehabilitation versus routine care
Wu et al., 2024 [21]	China	Prospective cluster-randomized controlled trial; ward rooms as the unit of randomization (8 clusters)	Oral cancer after extended resection and free-flap reconstruction; single centre, Peking University School and Hospital of Stomatology; recruited February–August 2022	126 enrolled; 121 analysed (62/59)	Early swallowing training from postoperative day 6	Routine nursing and swallowing-related education	Postoperative swallowing/oral function/quality of life	Postoperative day 15 and 1 month	Separate quantitative estimate: postoperative MASA-OC
Wang et al., 2019 [27]	Taiwan	Parallel randomized clinical trial with active control	Postoperative oral cancer patients after curative surgery	68 randomized; 30/30 analysed	Mouth-opening programme plus structured telephone support	Same mouth-opening programme without structured support	Mouth opening/trismus/adherence	1 and 3 months after discharge	Separate quantitative estimates: telephone support versus the same programme
Saghafi et al., 2026 [28]	Sweden	Single-blinded randomized controlled trial	Newly diagnosed HNC scheduled for curative RT ± chemotherapy; no baseline trismus	58 randomized; 25/27 assessed at 12 months	Preventive active and passive jaw training with JawTrainer	Standard care	Mouth opening/trismus/temporomandibular disorders	6 and 12 months after RT	Meta-analysis: mouth opening and trismus
Shanmugam and Pravenkumar, 2024 [40]	India	Randomized controlled trial	Oral cavity squamous cell carcinoma undergoing major resection	60 randomized; 30/30 analysed	Preoperative swallowing exercises plus standard postoperative rehabilitation	Standard postoperative rehabilitation alone	Swallowing prehabilitation before surgery	6 months after surgery	Separate quantitative estimate: prehabilitation added to postoperative rehabilitation

RT: radiotherapy; CRT: chemoradiotherapy; IMRT: intensity-modulated radiotherapy; HNC: head and neck cancer; HRQL: health-related quality of life; MIO: maximum interincisal opening; MMO: maximum mouth opening; TMD: temporomandibular disorders; MASA: Mann Assessment of Swallowing Ability; MASA-OC: Mann Assessment of Swallowing Ability-Oral Cancer; OPSE: oropharyngeal swallow efficiency; MDADI: MD Anderson Dysphagia Inventory; UW-QOL: University of Washington Quality of Life Questionnaire; FOIS: Functional Oral Intake Scale; PEG: percutaneous endoscopic gastrostomy; NMES: neuromuscular electrical stimulation; SLP: speech-language pathologist.

### 3.3. Risk of Bias

**Figure 2:**
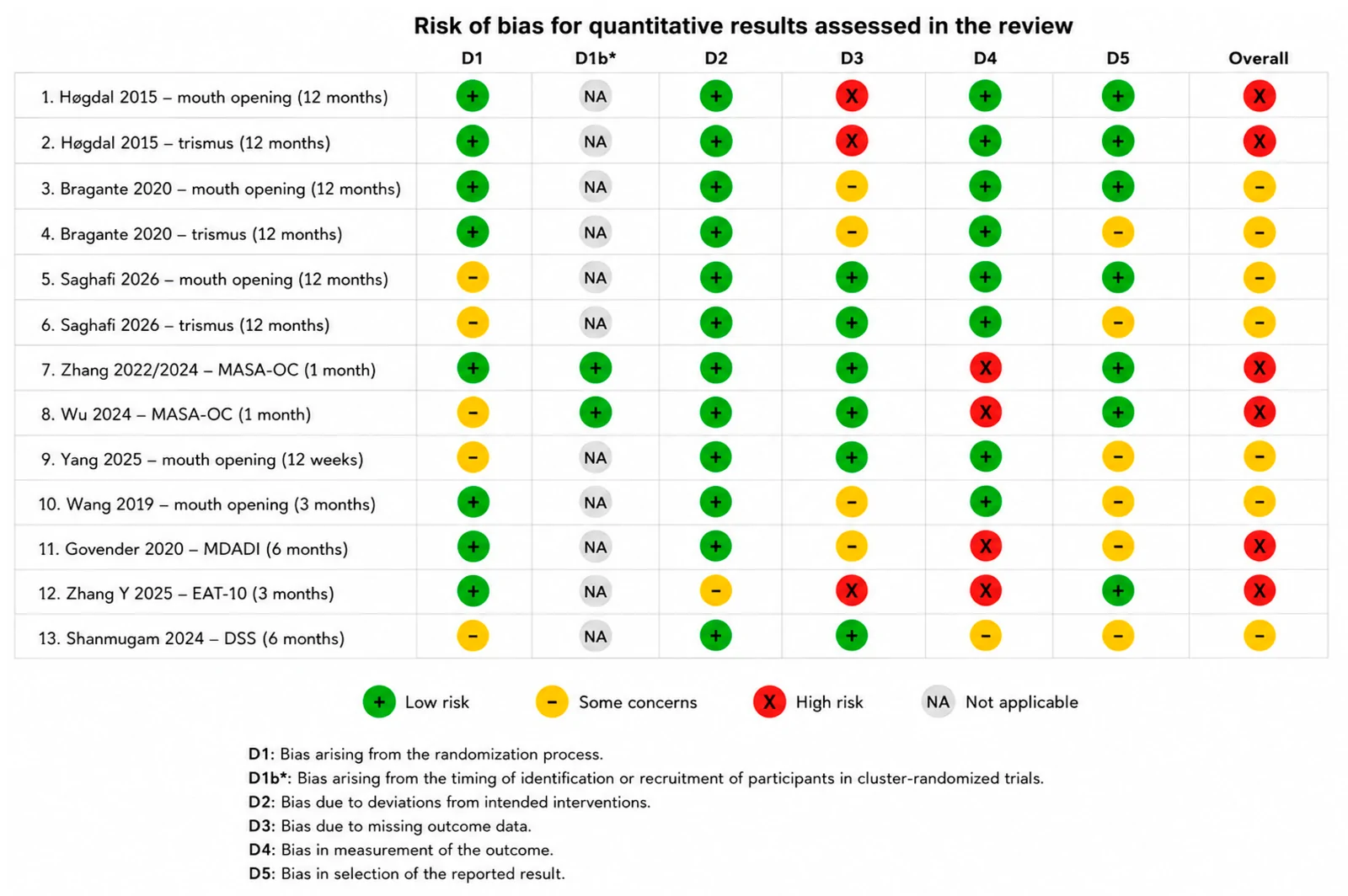
Risk-of-bias assessment for the quantitative results assessed in the review [20,21,22,23,24,27,28,32,36,39,40]. D1: bias arising from the randomization process; D1b*: bias arising from the identification or recruitment of participants in cluster-randomized trials; D2: bias due to deviations from intended interventions; D3: bias due to missing outcome data; D4: bias in measurement of the outcome; D5: bias in selection of the reported result; * D1b applies only to cluster-randomized trials.

Risk of bias was assessed for 13 quantitative results: six effect estimates included in the two meta-analyses and seven separately reported quantitative estimates. Seven results were judged as having some concerns and six as having high risk of bias; none was classified as low risk overall. The mouth-opening and trismus results from Høgdal et al. were judged to be at high risk because of missing outcome data. The corresponding results from Bragante et al. and Saghafi et al. raised some concerns, mainly related to missing outcome data, the randomization process, or selection of the reported result. The postoperative MASA-OC results from the Zhang and Wu trials were judged to be at high risk because the clinician-assessed outcome could have been influenced by knowledge of the assigned intervention. Among the additional separately reported estimates, Yang et al. [22], Wang et al. [27], and Shanmugam and Pravenkumar [40] raised some concerns, whereas Govender et al. [36] and Zhang Y et al. [39] were judged to be at high risk of bias. Domain-level judgements are shown in Figure 2, and the supporting reasons are provided in Supplementary Table S2.

### 3.4. Preventive Jaw Rehabilitation Versus Usual Care: Mouth Opening

**Figure 3:**
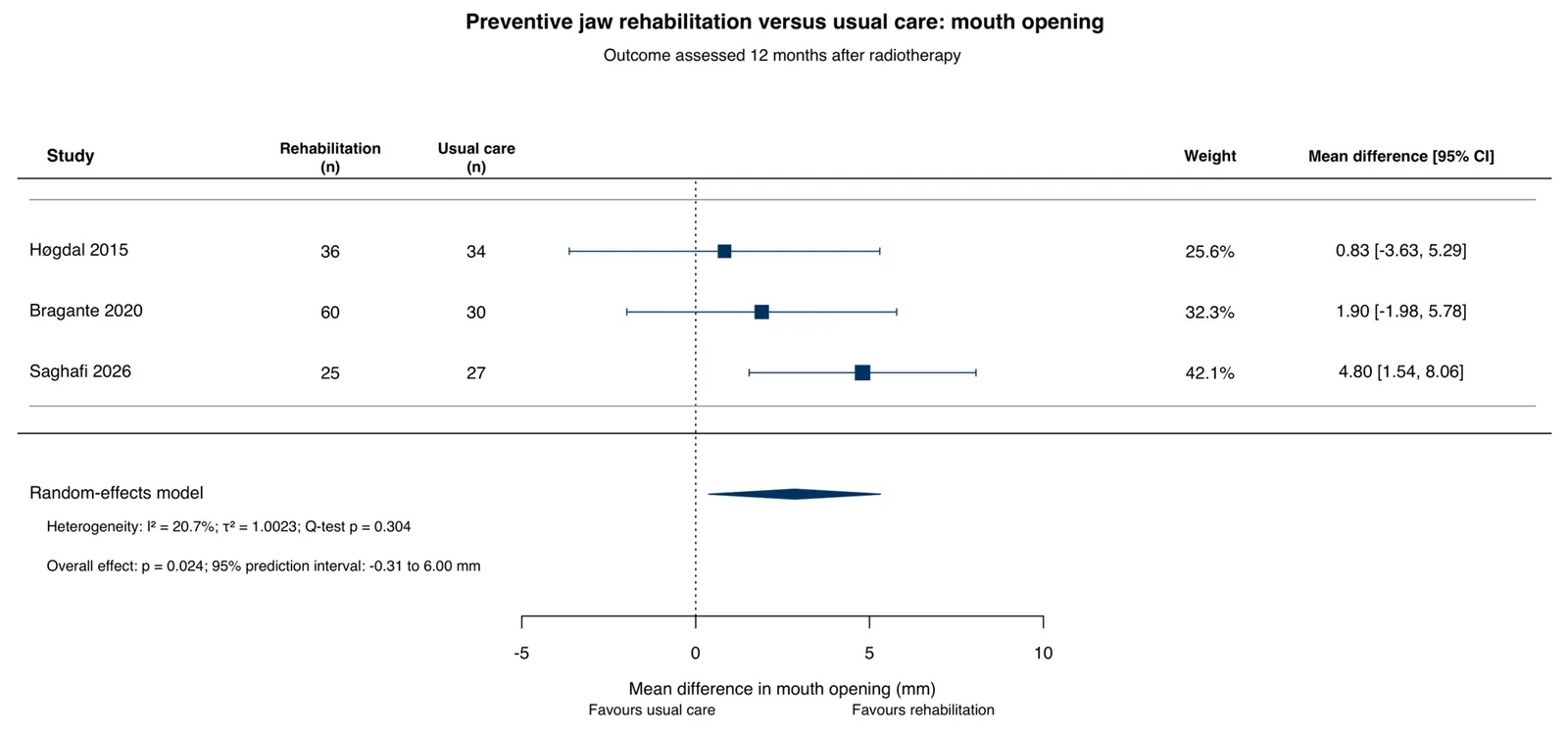
Effect of preventive jaw rehabilitation versus usual care on mouth opening [23,24,28]. Forest plot of the random-effects meta-analysis, expressed as mean difference in millimetres. Positive values favour rehabilitation. The pooled confidence interval and prediction interval use the conventional REML/Wald framework; the modified Hartung–Knapp sensitivity analysis is reported in the text.

Three independent trials comparing preventive jaw rehabilitation with usual care contributed to the mouth-opening meta-analysis [23,24,28]. All three trials contributed change-from-baseline estimates to the principal analysis. Høgdal et al. included 36 intervention and 34 control participants with complete measurements across the required assessments, whereas the Bragante et al. [24] estimate was based on the intention-to-treat GEE analysis of all randomized participants (60 combined intervention and 30 control). Saghafi et al. contributed 25 intervention and 27 control participants at 12 months. The pooled estimate favoured rehabilitation (MD = 2.85 mm). Under the primary conventional Wald inference, the 95% CI was 0.38 to 5.32 (*p* = 0.024). The modified Hartung–Knapp sensitivity analysis produced a wider interval that included no effect (95% CI −2.58 to 8.27; *p* = 0.152), indicating that statistical inference was sensitive to the method used. Between-study heterogeneity was low (I^2^ = 20.7%, τ^2^ = 1.0023; Q-test *p* = 0.304), and the conventional 95% prediction interval ranged from −0.31 to 6.00 mm. Given that it was estimated from only three trials, this prediction interval is highly uncertain and should be interpreted descriptively (Figure 3).

The direction of effect was consistent across the included trials. Sensitivity analyses in which each active arm of the three-arm Bragante trial was entered separately also favoured rehabilitation under conventional Wald inference (MD = 3.03 mm, 95% CI 0.58 to 5.48; and MD = 2.81 mm, 95% CI 0.06 to 5.56). The endpoint-only analysis produced a comparable estimate, although with lower precision (MD = 2.50 mm, 95% CI −0.51 to 5.51).

### 3.5. Preventive Jaw Rehabilitation Versus Usual Care: Trismus

**Figure 4:**
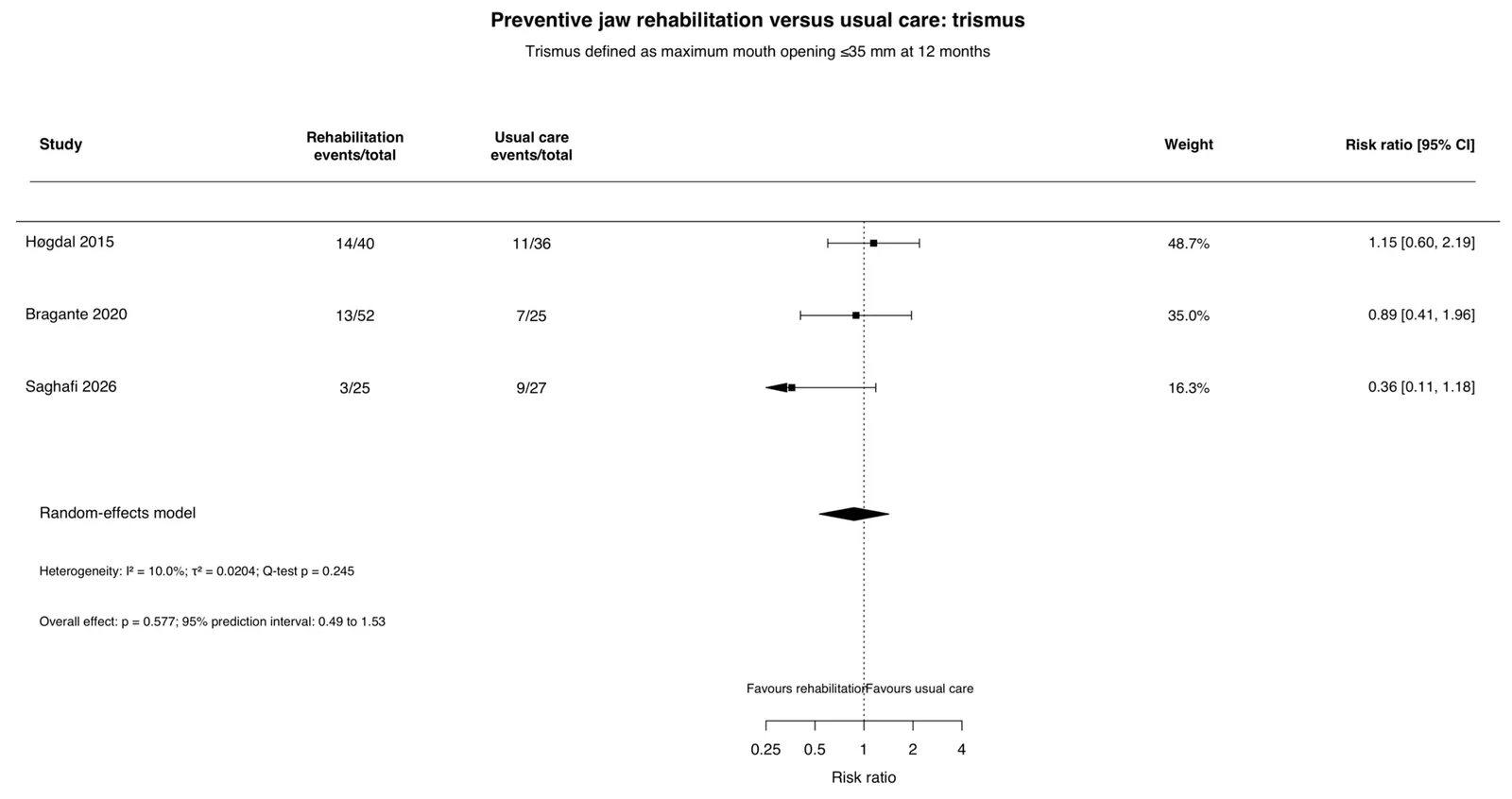
Effect of preventive jaw rehabilitation versus usual care on trismus [23,24,28]. Forest plot of the random-effects meta-analysis. Risk ratios below 1 favour rehabilitation. The pooled confidence interval and prediction interval use the conventional REML/Wald framework; the modified Hartung–Knapp sensitivity analysis is reported in the text.

Three independent trials comparing preventive jaw rehabilitation with usual care reported dichotomous trismus outcomes [23,24,28]. For Bragante et al. [24], the 12-month trismus analysis included participants with available outcome data (13/52 events in the combined intervention groups and 7/25 in the control group), rather than the full intention-to-treat sample used for continuous mouth opening. In Høgdal et al., the 12-month trismus analysis included all participants assessed at that visit (14/40 vs. 11/36), whereas the continuous mouth-opening analysis required complete measurements across the relevant assessments (36/34). The pooled estimate favoured rehabilitation but was imprecise and compatible with no effect (RR = 0.87). Under conventional Wald inference, the 95% CI was 0.53 to 1.42 (*p* = 0.577). The modified Hartung–Knapp sensitivity analysis yielded a wider interval that also included no effect (95% CI 0.25 to 2.98; *p* = 0.673). Individual point estimates were not uniformly aligned, as Høgdal et al. favoured usual care (RR = 1.15). Between-study heterogeneity was low (I^2^ = 10.0%, τ^2^ = 0.0204; Q-test *p* = 0.245), and the conventional 95% prediction interval ranged from 0.49 to 1.53. Given that it was estimated from only three trials, this prediction interval is highly uncertain and should be interpreted descriptively (Figure 4). Sensitivity analyses using each Bragante intervention arm separately or the 6-month Saghafi assessment did not materially change the direction of the result.

### 3.6. Postoperative Swallowing Rehabilitation After Oral-Cancer Surgery

Two sequential cluster-randomized cohorts comparing postoperative swallowing rehabilitation with routine care after oral-cancer surgery and free-flap reconstruction reported MASA-OC outcomes at 1 month [20,21,32]. Both were conducted at the same centre under the same registered protocol (ChiCTR2100050398), but recruitment periods did not overlap: Zhang et al. recruited from August 2021 to January 2022 using 18 ward-room clusters, whereas Wu et al. recruited a subsequent cohort from February to August 2022 using eight ward-room clusters. No participant contributed to both estimates; the datasets were therefore treated as participant-level independent. Published model-based estimates and standard errors were used for both cohorts. Both estimates favoured rehabilitation but were reported separately because the variance estimates were not sufficiently comparable for pooling. Zhang et al. [20] reported an adjusted between-group difference in change of 10.06 points (95% CI 7.68–12.44), whereas Wu et al. [21] reported a GEE group-by-time coefficient of 7.289 (SE = 1.621; 95% CI 4.11–10.47).

### 3.7. Additional Intervention Contrasts and Narrative Synthesis

Trials comparing early versus delayed rehabilitation, basic versus enhanced rehabilitation, the same programme with versus without additional support, or alternative active modalities or delivery strategies were analysed separately from rehabilitation-versus-usual-care trials. Several of these comparisons produced favourable estimates, including earlier initiation of mouth-opening exercises, telephone-supported mouth-opening rehabilitation, and context-specific swallowing interventions. The corresponding quantitative results are presented in Supplementary Table S1.

The remaining studies were synthesized narratively when differences in intervention contrast, clinical context, outcome, or available numerical data precluded pooling. These included voice and communication rehabilitation, post-laryngectomy rehabilitation, adherence and delivery-mode interventions, active-device comparisons, and active versus sham neuromuscular electrical stimulation. Companion and follow-up publications from the same randomized population were treated as reports of a single trial.

## 4. Discussion

This systematic review provides a comprehensive synthesis of 21 independent randomized trials evaluating rehabilitation across the spectrum of functional sequelae associated with head and neck cancer. The revised quantitative synthesis, organized according to the randomized intervention contrast, favoured greater mouth opening, although the small-sample-adjusted sensitivity interval included no effect. Separate postoperative swallowing estimates also favoured rehabilitation. Preventive jaw rehabilitation also produced a favourable pooled estimate for trismus, although the available evidence was less precise. Other trials addressed intervention timing, additional support, enhanced programmes, and alternative active modalities, extending the evidence beyond the comparisons included in the meta-analyses.

The mouth-opening meta-analysis favoured preventive jaw rehabilitation compared with usual care, with a pooled mean difference of 2.85 mm and low between-study heterogeneity. The direction of effect was consistent across the included trials and analyses using either active arm of the Bragante trial. However, while the conventional Wald confidence interval excluded no effect, the modified Hartung–Knapp sensitivity interval included no effect, showing that the inferential conclusion was sensitive to the method used. The conventional prediction interval also included the possibility of no effect and is highly uncertain because it was estimated from only three trials. Taken together, the estimates suggest a possible benefit but remain insufficiently precise for a firm inferential conclusion. Structured jaw mobilization may counteract treatment-related stiffness and help preserve mandibular mobility. Improvements in mouth opening may facilitate mastication, oral hygiene, speech, dental care, and routine clinical examination, particularly in patients exposed to radiotherapy-related fibrosis or postoperative scar restriction [11,47,48].

The pooled estimate for trismus also favoured preventive jaw rehabilitation but was imprecise and compatible with no effect, with low between-study heterogeneity. Both the conventional and modified Hartung–Knapp confidence intervals included no effect, with the latter being substantially wider. The conventional prediction interval also included no effect and is highly uncertain because it was estimated from only three trials. The available evidence therefore remains insufficiently precise to confirm the magnitude of the preventive benefit; moreover, the Høgdal point estimate favoured usual care (RR = 1.15), despite the pooled estimate favouring rehabilitation. Preserving mandibular mobility before established fibrosis develops may be more achievable than reversing long-standing restriction. Further trials with larger samples, consistent diagnostic thresholds, and detailed adherence reporting are needed to define the preventive effect more precisely [11,47,48,49].

Postoperative swallowing rehabilitation produced favourable estimates in MASA-OC scores after oral-cancer surgery and free-flap reconstruction. Both cohort-specific estimates favoured rehabilitation: the adjusted between-group difference in change was 10.06 points (95% CI 7.68–12.44) in Zhang et al. [20], and the GEE group-by-time coefficient was 7.29 points (95% CI 4.11–10.47) in Wu et al. [21]. The estimates were not pooled because their variance structures could not be established as sufficiently comparable. Early, structured swallowing rehabilitation may therefore improve functional recovery in this specific postoperative population. The result applies to a defined surgical context and objective clinical measure, while preventive exercise during radiotherapy, prehabilitation, and rehabilitation of established dysphagia were evaluated through distinct comparisons. Presenting these settings separately provides a more clinically informative account of the available evidence.

The trials analysed separately provide complementary information on how rehabilitation programmes may be optimized. Favourable individual estimates were reported for earlier initiation of mouth-opening exercises [22], the addition of structured telephone support to a mouth-opening programme [27], and selected swallowing interventions delivered before, during, or after oncological treatment [36,39,40]. These studies evaluated timing, additional components, or delivery strategies rather than the same treatment contrast as the principal meta-analyses and were therefore presented separately. The broad clinical scope of the review identified substantial diversity in intervention content, comparator conditions, treatment timing, supervision, tumour site, and oncological treatment. Reorganizing the analyses according to the randomized contrast reduced conceptual heterogeneity and produced clinically interpretable estimates for specific rehabilitation questions. The estimates are therefore most applicable to the specific functional deficit, treatment stage, and therapeutic objective evaluated.

The result-specific RoB 2 assessment identified some concerns for seven results and high risk of bias for six results. The high-risk judgements for the Høgdal outcomes arose from missing follow-up data, whereas those for postoperative swallowing were related to clinician-assessed MASA-OC scores in non-blinded trials. The Bragante and Saghafi results raised some concerns but retained low-risk judgements for outcome measurement. Among the additional separately reported estimates, Yang et al. [22], Wang et al. [27], and Shanmugam and Pravenkumar [40] raised some concerns, whereas Govender et al. [36] and Zhang Y et al. [39] were judged to be at high risk of bias. The direction of effect remained consistent within the mouth-opening analysis and across the postoperative swallowing estimates, although the magnitude of these estimates should be interpreted in light of the identified methodological limitations.

Preventive jaw rehabilitation produced favourable estimates for mandibular mobility, while postoperative swallowing rehabilitation also favoured functional recovery after oral-cancer surgery and free-flap reconstruction. The favourable direction of the trismus estimate warrants further investigation of preventive jaw exercise during oncological treatment. Preventive jaw and postoperative swallowing rehabilitation may be considered according to the patient’s functional presentation and treatment stage, with physiotherapists, speech-language pathologists, and other rehabilitation professionals contributing to multidisciplinary head and neck cancer care.

Future research should focus on improving methodological consistency across rehabilitation trials. Larger multicentre randomized controlled trials with clearly defined comparator conditions are required to refine the optimal timing, intensity, therapeutic components, and delivery of rehabilitation interventions [50]. Standardization of outcome measures would facilitate comparison between studies and allow more precise quantitative syntheses. Furthermore, future investigations should evaluate long-term functional recovery, patient-reported outcomes, adherence to rehabilitation programmes, and cost-effectiveness to better define the role of rehabilitation within contemporary head and neck cancer management [11,49].

The present review has several limitations that should be considered when interpreting the findings. First, the broad scope of the review encompassed differences in participant characteristics, rehabilitation interventions, comparator conditions, treatment timing, programme duration, and outcome measures. Restricting each meta-analysis to a homogeneous intervention contrast improved clinical interpretability, although it reduced the number of trials contributing to each pooled estimate.

Second, several intervention contrasts were represented by individual randomized trials, and the two meta-analyses each included three independent trials. The use of different assessment instruments and follow-up periods limited quantitative pooling across some swallowing, voice, communication, and adherence-related outcomes. These findings were therefore retained through separate quantitative estimates and narrative synthesis. The restriction to publications in English or Spanish may also have resulted in the exclusion of potentially relevant evidence published in other languages. With only three trials in each meta-analysis, estimation of τ^2^, small-sample-adjusted inference, and prediction intervals is intrinsically unstable; the modified Hartung–Knapp analyses were therefore used to assess robustness rather than as definitive inferential tests.

Another limitation concerns the risk of bias of the quantitative results assessed in this review. Seven result-specific assessments raised some concerns and six were judged to be at high risk, mainly because of missing outcome data or potential influence of allocation knowledge on clinician-assessed outcomes. These issues reduce confidence in the precision of the pooled and separately reported estimates and should be considered when interpreting their magnitude. In addition, the small number of trials available for each outcome precluded a reliable assessment of publication bias.

## 5. Conclusions

Some targeted rehabilitation interventions produced favourable functional estimates in adults with head and neck cancer. Compared with usual care, preventive jaw rehabilitation favoured greater mouth opening, but the small-sample-adjusted confidence interval included no effect. The pooled estimate for trismus also favoured rehabilitation, although further trials are needed to determine the magnitude of this preventive effect more precisely. Postoperative swallowing rehabilitation favoured higher MASA-OC scores in both sequential cohorts after oral-cancer surgery and free-flap reconstruction.

The most consistent favourable estimates were observed for mandibular mobility and postoperative swallowing function, while separate trials provided additional favourable evidence regarding intervention timing, therapeutic enhancement, and delivery support. Rehabilitation strategies should be selected according to the functional deficit and clinical context within multidisciplinary head and neck cancer care.

## Data Availability

The data supporting the findings of this study are available within the article and its Supplementary Materials.

## Supplementary Materials

The following supporting information can be downloaded at: https://www.mdpi.com/article/10.3390/medicina62091648/s1, Supplementary Material S1: Complete Electronic Search Strategies; Supplementary Material S2: PRISMA 2020 Checklist; Table S1: Individual effect estimates for intervention contrasts not included in meta-analysis; Table S2: Result-specific risk-of-bias assessments for quantitative results. Reference [51] is cited in the supplementary materials.
