# Supplementary material for: Rehabilitation and Physiotherapy for Functional Sequelae in Adults with Head and Neck Cancer: A Systematic Review and Meta-Analysis
Source: Medicina (Kaunas). 2026 Aug 28;62(9):1648. doi: 10.3390/medicina62091648 (PMC13609679; doi:10.3390/medicina62091648)
Supplement: Supplementary file 1 [file medicina-62-01648-s001.zip › Supplementary Material S1.pdf]

Supplementary Material S1. Complete Electronic Search Strategies

Rehabilitation and Physiotherapy for Functional Sequelae in Adults with Head and Neck Cancer: a Systematic Review and Meta-analysis

Search overview

PubMed/MEDLINE, Embase, the Cochrane Library (CENTRAL), and PEDro were searched from database inception through 10 June 2026. Search concepts combined terms for head and neck cancer with terms for rehabilitation and physiotherapy. Validated randomized-trial filters were applied in PubMed/MEDLINE and Embase. CENTRAL was searched without an additional randomized-trial filter because it is a trial-focused database, and PEDro results were restricted to clinical trials using the platform method filter. No date limits were applied. The record counts below correspond to the final search sets retrieved on 10 June 2026 before deduplication.

The reference lists of included studies and relevant systematic reviews were also screened manually. This citation searching identified no additional records (n = 0).

Summary of electronic database searches

Database / platform	Coverage	Last searched	Records retrieved
PubMed/MEDLINE	Inception to 10 June 2026	10 June 2026	380
Embase (Embase.com)	Inception to 10 June 2026	10 June 2026	569
Cochrane Library (CENTRAL)	Inception to 10 June 2026	10 June 2026	587
PEDro	Inception to 10 June 2026	10 June 2026	0
**Total**			**1,536**

PubMed/MEDLINE

Interface: PubMed. The randomized-trial filter is reproduced in full in line #3 (Cochrane Highly Sensitive Search Strategy, sensitivity-maximizing PubMed format).

Line	Search strategy
#1	("Head and Neck Neoplasms"[Mesh] OR "head and neck cancer*"[tiab] OR "head and neck neoplasm*"[tiab] OR "oral cancer*"[tiab] OR "oral neoplasm*"[tiab] OR "oropharyngeal cancer*"[tiab] OR "nasopharyngeal cancer*"[tiab] OR "hypopharyngeal cancer*"[tiab] OR "laryngeal cancer*"[tiab] OR "mouth neoplasm*"[tiab] OR "tongue cancer*"[tiab])
#2	("Rehabilitation"[Mesh] OR "Physical Therapy Modalities"[Mesh] OR rehabilitation[tiab] OR physiotherapy[tiab] OR "physical therapy"[tiab] OR exercise*[tiab] OR "exercise therapy"[tiab] OR "therapeutic exercise"[tiab] OR "manual therapy"[tiab] OR stretching[tiab] OR "resistance training"[tiab] OR telerehabilitation[tiab] OR "home exercise"[tiab] OR "physical rehabilitation"[tiab] OR "functional training"[tiab])
#3	(randomized controlled trial[pt] OR controlled clinical trial[pt] OR randomized[tiab] OR placebo[tiab] OR drug therapy[sh] OR randomly[tiab] OR trial[tiab] OR groups[tiab]) NOT (animals[mh] NOT humans[mh])
#4	#1 AND #2 AND #3

Final search set: #1 AND #2 AND #3

Records retrieved on 10 June 2026: 380

PubMed field tags: [Mesh], Medical Subject Heading; [tiab], title/abstract; [pt], publication type; [sh], subheading; [mh], MeSH term.

Embase

Interface: Embase.com. Lines F1-F35 reproduce the Cochrane Highly Sensitive Search Strategy for identifying randomized trials in Embase (2023 revision, Embase.com format).

Line	Search strategy
A	('head and neck cancer'/exp OR 'oral cancer':ti,ab OR 'head and neck cancer':ti,ab OR 'oropharyngeal cancer':ti,ab OR 'nasopharyngeal cancer':ti,ab OR 'laryngeal cancer':ti,ab)
B	('rehabilitation'/exp OR 'physical therapy'/exp OR rehabilitation:ti,ab OR physiotherapy:ti,ab OR 'physical therapy':ti,ab OR exercise*:ti,ab OR 'manual therapy':ti,ab)
F1	'randomized controlled trial'/exp
F2	'controlled clinical trial'/de
F3	random*:ti,ab,tt
F4	'randomization'/de
F5	'intermethod comparison'/de
F6	placebo:ti,ab,tt
F7	(compare:ti,tt OR compared:ti,tt OR comparison:ti,tt)
F8	((evaluated:ab OR evaluate:ab OR evaluating:ab OR assessed:ab OR assess:ab) AND (compare:ab OR compared:ab OR comparing:ab OR comparison:ab))
F9	(open NEXT/1 label):ti,ab,tt
F10	((double OR single OR doubly OR singly) NEXT/1 (blind OR blinded OR blindly)):ti,ab,tt
F11	'double blind procedure'/de
F12	(parallel NEXT/1 group*):ti,ab,tt
F13	(crossover:ti,ab,tt OR 'cross over':ti,ab,tt)
F14	((assign* OR match OR matched OR allocation) NEAR/6 (alternate OR group OR groups OR intervention OR interventions OR patient OR patients OR subject OR subjects OR participant OR participants)):ti,ab,tt
F15	(assigned:ti,ab,tt OR allocated:ti,ab,tt)
F16	(controlled NEAR/8 (study OR design OR trial)):ti,ab,tt
F17	(volunteer:ti,ab,tt OR volunteers:ti,ab,tt)
F18	'human experiment'/de
F19	trial:ti,tt
F20	F1 OR F2 OR F3 OR F4 OR F5 OR F6 OR F7 OR F8 OR F9 OR F10 OR F11 OR F12 OR F13 OR F14 OR F15 OR F16 OR F17 OR F18 OR F19
F21	(((random* NEXT/1 sampl* NEAR/8 ('cross section*' OR questionnaire* OR survey OR surveys OR database OR databases)):ti,ab,tt) NOT ('comparative study'/de OR 'controlled study'/de OR 'randomised controlled':ti,ab,tt OR 'randomized controlled':ti,ab,tt OR 'randomly assigned':ti,ab,tt))
F22	('cross-sectional study'/de NOT ('randomized controlled trial'/exp OR 'controlled clinical study'/de OR 'controlled study'/de OR 'randomised controlled':ti,ab,tt OR 'randomized controlled':ti,ab,tt OR 'control group':ti,ab,tt OR 'control groups':ti,ab,tt))
F23	('case control*':ti,ab,tt AND random*:ti,ab,tt NOT ('randomised controlled':ti,ab,tt OR 'randomized controlled':ti,ab,tt))
F24	('systematic review':ti,tt NOT (trial:ti,tt OR study:ti,tt))

Line	Search strategy
F25	(nonrandom*:ti,ab,tt NOT random*:ti,ab,tt)
F26	'random field*':ti,ab,tt
F27	('random cluster' NEAR/4 sampl*):ti,ab,tt
F28	(review:ab AND review:it) NOT trial:ti,tt
F29	('we searched':ab AND (review:ti,tt OR review:it))
F30	'update review':ab
F31	(databases NEAR/5 searched):ab
F32	((rat:ti,tt OR rats:ti,tt OR mouse:ti,tt OR mice:ti,tt OR swine:ti,tt OR porcine:ti,tt OR murine:ti,tt OR sheep:ti,tt OR lambs:ti,tt OR pigs:ti,tt OR piglets:ti,tt OR rabbit:ti,tt OR rabbits:ti,tt OR cat:ti,tt OR cats:ti,tt OR dog:ti,tt OR dogs:ti,tt OR cattle:ti,tt OR bovine:ti,tt OR monkey:ti,tt OR monkeys:ti,tt OR trout:ti,tt OR marmoset*:ti,tt) AND 'animal experiment'/de)
F33	('animal experiment'/de NOT ('human experiment'/de OR 'human'/de))
F34	F21 OR F22 OR F23 OR F24 OR F25 OR F26 OR F27 OR F28 OR F29 OR F30 OR F31 OR F32 OR F33
F35	F20 NOT F34
C	A AND B AND F35

Final search set: A AND B AND F35

Records retrieved on 10 June 2026: 569

Embase.com syntax: /exp, exploded Emtree term; /de, Emtree descriptor; :ti, title; :ab, abstract; :tt, original non-English title; :it, item type.

Cochrane Library (CENTRAL)

Database: Cochrane Central Register of Controlled Trials (CENTRAL), searched through the Cochrane Library. The search was run in CENTRAL without an additional randomized-trial methodological filter.

Line	Search strategy
#1	("head and neck cancer" OR "head and neck neoplasm*" OR "oral cancer" OR "oropharyngeal cancer" OR "nasopharyngeal cancer" OR "laryngeal cancer")
#2	(rehabilitation OR physiotherapy OR "physical therapy" OR "exercise therapy" OR exercise* OR "manual therapy")
#3	#1 AND #2

Final search set: #1 AND #2, restricted to CENTRAL

Records retrieved on 10 June 2026: 587

No separate randomized-trial filter was applied in CENTRAL because CENTRAL is a trial-focused database and such filters are not recommended for this source.

PEDro

Database: Physiotherapy Evidence Database (PEDro). Search terms were combined and results were restricted to clinical trials using the PEDro method filter.

Line	Search strategy
1	Search term: "head and neck cancer"
2	Search term: rehabilitation

Line	Search strategy
3	Combination: "head and neck cancer" AND rehabilitation
4	Method filter: clinical trial

Final search set: "head and neck cancer" AND rehabilitation; Method = clinical trial

Records retrieved on 10 June 2026: 0

Methodological note

The randomized-trial filters reproduced above follow the Cochrane Highly Sensitive Search Strategies for PubMed/MEDLINE and Embase. CENTRAL was not filtered by study design because Cochrane guidance recommends against applying randomized-trial filters to CENTRAL.

Filter source: Lefebvre C, Glanville J, Briscoe S, et al. Chapter 4: Searching for and selecting studies. In: Higgins JPT, Thomas J, Chandler J, et al., editors. Cochrane Handbook for Systematic Reviews of Interventions, version 6.5.1. Cochrane; 2025.
