# Supplementary material for: Rehabilitation and Physiotherapy for Functional Sequelae in Adults with Head and Neck Cancer: A Systematic Review and Meta-Analysis
Source: Medicina (Kaunas). 2026 Aug 28;62(9):1648. doi: 10.3390/medicina62091648 (PMC13609679; doi:10.3390/medicina62091648)
Supplement: Supplementary file 1 [file medicina-62-01648-s001.zip › Supplementary Table S1.pdf]

Supplementary Table S1. Individual effect estimates for intervention contrasts not included in meta-analysis

Effects are presented using the scale and effect measure selected for each randomized contrast. Continuous effects were oriented so that positive values favour rehabilitation where indicated; risk ratios below 1 favour rehabilitation for adverse outcomes.

Study	Domain	Randomized contrast	Outcome	Follow-up	Effect estimate (95% CI)	Reason not pooled
Yang 2025	Jaw opening	Early versus delayed	Maximum interincisal opening	12 weeks post-surgery / 3 weeks after end of RT	MD (mm): 3.61 (3.03 to 4.19)	Timing contrast
Wang 2019	Jaw opening	Support versus same programme	Maximum interincisal opening	3 months after discharge vs pre-operation	MD (mm): 10.30 (8.23 to 12.38)	Telephone-support contrast
Messing 2017	Jaw opening (secondary)	Enhanced versus basic	Incisal opening	24 months post-CRT	MD (mm): 4.60 (0.30 to 8.90)	Both groups received active jaw care
Carnaby-Mann 2012	Swallowing	Rehabilitation versus usual care	MASA	CRT completion / 6 weeks	Hedges g: 0.41 (-0.34 to 1.16)	Single clinically homogeneous trial in CRT context
Govender 2020	Swallowing-related quality of life	Rehabilitation versus usual care	MDADI composite	6 months after treatment	Hedges g: 0.59 (-0.21 to 1.40)	Feasibility prehabilitation trial
Zhang Y 2025	Swallowing	Rehabilitation versus usual care	EAT-10	3 months post-treatment	MD, sign reversed: 3.88 (1.33 to 6.43)	Particle-therapy context and model-based scale-specific effect
Tuomi 2022 / Petersson 2023	Established dysphagia	Enhanced versus basic	Overall Swallowing Performance Scale	8-week follow-up	Hedges g, sign reversed: 0.28 (-0.27 to 0.83)	One independent HLE trial
Lazarus 2014	Swallowing	Enhanced versus basic	OPSE	Post-treatment after 6 weeks of exercise / 10 weeks post-RT	Hedges g: -0.43 (-1.42 to 0.56)	Active comparator and distinct clinical context
Zhang trial (2022/2024 reports)	Postoperative swallowing	Rehabilitation versus usual care	MASA-OC total score	1 month after surgery	Adjusted difference in change: 10.06 (7.68 to 12.44)	Variance estimate not sufficiently comparable with Wu 2024 for pooling
Wu 2024	Postoperative swallowing	Rehabilitation versus usual care	MASA-OC total score	1 month after surgery	GEE group-by-time β: 7.29 (4.11 to 10.47)	Variance estimate not sufficiently comparable with Zhang trial for pooling
Wang 2019	Trismus	Support versus same programme	Trismus prevalence	3 months after discharge	RR: 0.70 (0.56 to 0.89)	Telephone-support contrast
Carnaby-Mann 2012	Swallowing/oral intake	Rehabilitation versus usual care	Nonoral feeding	CRT completion / 6 weeks	RR: 0.58 (0.18 to 1.85)	Preventive CRT context
Wu 2024	Postoperative swallowing	Rehabilitation versus usual care	Dysphagia prevalence	1 month after surgery	RR: 0.34 (0.19 to 0.61)	Not pooled with CRT or prehabilitation outcomes
Shanmugam 2024	Swallowing/oral intake	Enhanced versus basic	Poor oral intake	6 months after surgery	RR: 0.08 (0.00 to 1.31)	Prehabilitation added to postoperative rehabilitation

Abbreviations: CRT, chemoradiotherapy; EAT-10, Eating Assessment Tool-10; GEE, generalized estimating equations; HLE, head-lift exercise; MASA, Mann Assessment of Swallowing Ability; MASA-OC, Mann Assessment of Swallowing Ability-Oral Cancer; MD, mean difference; MDADI, MD Anderson Dysphagia Inventory; OPSE, oropharyngeal swallow efficiency; RR, risk ratio; RT, radiotherapy.
