# Supplementary material for: Rehabilitation and Physiotherapy for Functional Sequelae in Adults with Head and Neck Cancer: A Systematic Review and Meta-Analysis
Source: Medicina (Kaunas). 2026 Aug 28;62(9):1648. doi: 10.3390/medicina62091648 (PMC13609679; doi:10.3390/medicina62091648)
Supplement: Supplementary file 1 [file medicina-62-01648-s001.zip › Supplementary Table S2.pdf]

Supplementary Table S2. Result-specific risk-of-bias assessments for quantitative results

The effect of interest was the effect of assignment to intervention. Individually randomized trials were assessed using RoB 2; cluster-randomized trials were assessed using the cluster-specific version. Assessments shown here are the consensus judgements of M.L.-B. and C.F.-M.

1. Høgdal et al., 2015: Maximal interincisor distance (12 months after radiotherapy)

Comparison: Preventive jaw rehabilitation + usual care vs usual care. Tool: RoB 2 for individually randomized parallel-group trials.

Domain	Judgement	Supporting reason
D1. Randomization process	Low risk	Central computer randomization by the Copenhagen Trial Unit, stratified by surgery; no important baseline imbalance attributable to randomization.
D1b. Identification/recruitment in cluster trials	Not applicable	Individually randomized trial.
D2. Deviations from intended interventions	Low risk	No evidence of trial-induced deviations that would compromise the effect of assignment; participants were analysed according to randomized groups.
D3. Missing outcome data	High risk	The primary analysis included only participants completing all three assessments. Missingness was substantial and included recurrence, poor condition and death; extreme-case imputations changed statistical significance.
D4. Measurement of the outcome	Low risk	Mouth opening was measured directly using a standardized scale by blinded assessors who were not involved in the intervention.
D5. Selection of the reported result	Low risk	Mouth opening and the 5- and 12-month assessments were specified, and unadjusted, adjusted and missing-data sensitivity analyses were reported.
Overall risk of bias	High risk	High risk arising from missing outcome data.

2. Høgdal et al., 2015: Trismus (maximal mouth opening ≤35 mm) (12 months after radiotherapy)

Comparison: Preventive jaw rehabilitation + usual care vs usual care. Tool: RoB 2 for individually randomized parallel-group trials.

Domain	Judgement	Supporting reason
D1. Randomization process	Low risk	Central computer randomization by the Copenhagen Trial Unit, stratified by surgery; no important baseline imbalance attributable to randomization.
D1b. Identification/recruitment in cluster trials	Not applicable	Individually randomized trial.
D2. Deviations from intended interventions	Low risk	No evidence of trial-induced deviations that would compromise the effect of assignment.
D3. Missing outcome data	High risk	The 12-month result was based on participants with available follow-up. Missingness was substantial and included recurrence, poor condition and death, which may be related to the true outcome.
D4. Measurement of the outcome	Low risk	Trismus was derived from a blinded objective measurement of mouth opening using the prespecified ≤35 mm threshold.
D5. Selection of the reported result	Low risk	The trismus threshold was specified in the methods and the 12-month result was reported.
Overall risk of bias	High risk	High risk arising from missing outcome data.

3. Bragante et al., 2020: Maximum mouth opening (12 months after radiotherapy)

Comparison: Combined active jaw rehabilitation arms vs usual care. Tool: RoB 2 for individually randomized parallel-group trials.

Domain	Judgement	Supporting reason
D1. Randomization process	Low risk	A statistician generated the random sequence; sealed, numbered, opaque envelopes were handled by an independent nurse; groups were comparable at baseline.
D1b. Identification/recruitment in cluster trials	Not applicable	Individually randomized trial.
D2. Deviations from intended interventions	Low risk	Participants and the trainer were aware of allocation, but no trial-induced deviations were identified; all randomized participants were included in the GEE intention-to-treat analysis.
D3. Missing outcome data	Some concerns	The GEE analysis included all randomized participants under a missing-at-random assumption, but the plausibility of this assumption for the 12-month missing outcomes was not fully demonstrated.
D4. Measurement of the outcome	Low risk	Mouth opening was measured with calibrated digital calipers by a blinded physiotherapist.
D5. Selection of the reported result	Low risk	Maximum mouth opening was the primary endpoint; the planned assessment times and primary unadjusted analysis were reported.
Overall risk of bias	Some concerns	Some concerns arising from missing outcome data.

4. Bragante et al., 2020: Trismus (maximum mouth opening ≤35 mm) (12 months after radiotherapy)

Comparison: Combined active jaw rehabilitation arms vs usual care. Tool: RoB 2 for individually randomized parallel-group trials.

Domain	Judgement	Supporting reason
D1. Randomization process	Low risk	A statistician generated the random sequence; sealed, numbered, opaque envelopes were handled by an independent nurse; groups were comparable at baseline.
D1b. Identification/recruitment in cluster trials	Not applicable	Individually randomized trial.
D2. Deviations from intended interventions	Low risk	No trial-induced deviations likely to bias the effect of assignment were identified.
D3. Missing outcome data	Some concerns	Some 12-month outcome data were unavailable and the relationship between missingness and the true trismus status was not fully assessed.
D4. Measurement of the outcome	Low risk	Trismus was derived from blinded objective mouth-opening measurements using the stated ≤35 mm threshold.
D5. Selection of the reported result	Some concerns	Trismus was a secondary dichotomized outcome and no sufficiently detailed prespecified analysis plan was available to rule out selection among eligible analyses.
Overall risk of bias	Some concerns	Some concerns arising from missing outcome data and selection of the reported result.

5. Saghafi et al., 2026: Maximal incisal opening (12 months after radiotherapy)

Comparison: Preventive jaw training vs standard care. Tool: RoB 2 for individually randomized parallel-group trials.

Domain	Judgement	Supporting reason
D1. Randomization process	Some concerns	Computerized Pocock sequential allocation was reported, but allocation concealment and implementation of the sequence were not described in sufficient detail.
D1b. Identification/recruitment in cluster trials	Not applicable	Individually randomized trial.
D2. Deviations from intended interventions	Low risk	No trial-induced deviations likely to bias the effect of assignment were identified; the primary analysis followed the randomized groups.
D3. Missing outcome data	Low risk	Outcome data were available for 52 of 58 participants; multiple imputation was used for the primary endpoint and dropout analyses did not identify baseline differences.
D4. Measurement of the outcome	Low risk	Mouth opening was measured directly by calibrated examiners blinded to allocation.
D5. Selection of the reported result	Low risk	Mouth opening was identified as the primary measure; both follow-up times and ITT, complete-case and per-protocol analyses were reported.
Overall risk of bias	Some concerns	Some concerns arising from the randomization process.

6. Saghafi et al., 2026: Trismus (maximal incisal opening ≤35 mm) (12 months after radiotherapy)

Comparison: Preventive jaw training vs standard care. Tool: RoB 2 for individually randomized parallel-group trials.

Domain	Judgement	Supporting reason
D1. Randomization process	Some concerns	Computerized Pocock sequential allocation was reported, but allocation concealment and implementation of the sequence were not described in sufficient detail.
D1b. Identification/recruitment in cluster trials	Not applicable	Individually randomized trial.
D2. Deviations from intended interventions	Low risk	No trial-induced deviations likely to bias the effect of assignment were identified; rescue jaw training followed clinical criteria and was available irrespective of original group.
D3. Missing outcome data	Low risk	Trismus status was available for 52 of 58 participants, with few and balanced losses and no baseline differences identified in dropout analyses.
D4. Measurement of the outcome	Low risk	Trismus was derived from blinded objective measurement of mouth opening using the stated ≤35 mm threshold.
D5. Selection of the reported result	Some concerns	Trismus incidence was a secondary result and no sufficiently detailed prespecified analysis plan was available to rule out selection among time points and related diagnostic outcomes.
Overall risk of bias	Some concerns	Some concerns arising from the randomization process and selection of the reported result.

7. Zhang trial (2022/2024 reports): MASA-OC total score (1 month after surgery)

Comparison: Personalized postoperative oral/swallowing rehabilitation vs routine care. Tool: RoB 2 for cluster-randomized trials.

Domain	Judgement	Supporting reason
D1. Randomization process	Low risk	Eighteen ward rooms were randomly allocated using SPSS; room assignment was performed by nurses unaware of group allocation; baseline characteristics were comparable.
D1b. Identification/recruitment in cluster trials	Low risk	Patients were assigned to rooms by nurses unaware of the room allocation and were informed of their group after enrolment, limiting differential identification or recruitment.
D2. Deviations from intended interventions	Low risk	No important deviations from the assigned strategies were reported, and analyses retained the randomized comparison.
D3. Missing outcome data	Low risk	Sixty-eight of 75 allocated participants completed the 1-month assessment (34/34 among those completing follow-up); losses were few and balanced between groups.
D4. Measurement of the outcome	High risk	MASA-OC is a clinician-rated outcome. The specialist swallowing nurse was involved in intervention delivery and outcome assessment, and the trial was not blinded; knowledge of allocation could plausibly influence scoring.
D5. Selection of the reported result	Low risk	MASA-OC was the primary outcome, day 15 and 1-month assessments were prespecified, and the mixed-effects model accounted for room clustering and baseline score.
Overall risk of bias	High risk	High risk arising from measurement of the outcome.

8. Wu et al., 2024: MASA-OC total score (1 month after surgery)

Comparison: Early postoperative swallowing training vs routine care. Tool: RoB 2 for cluster-randomized trials.

Domain	Judgement	Supporting reason
D1. Randomization process	Some concerns	Eight ward rooms were randomized using SPSS and assigning nurses were unaware of allocation, but the published GEE analysis did not clearly state whether ward-room clustering was incorporated.
D1b. Identification/recruitment in cluster trials	Low risk	A nurse unaware of room allocation assigned rooms before investigators informed participants of their group, limiting differential recruitment.
D2. Deviations from intended interventions	Low risk	No important deviations from the assigned intervention strategies were reported.
D3. Missing outcome data	Low risk	Only two participants were lost after discharge; the amount of missing data was too small to materially affect the result.
D4. Measurement of the outcome	High risk	MASA-OC was assessed by nursing staff in a non-blinded trial while specialist nurses delivered and supervised the intervention; the clinician-rated score could plausibly be influenced by knowledge of allocation.
D5. Selection of the reported result	Low risk	MASA-OC was the main outcome, the day 15 and 1-month assessments were prespecified, and both time-specific and GEE results were reported.
Overall risk of bias	High risk	High risk arising from measurement of the outcome.

9. Yang et al., 2025: Maximum interincisal opening (12 weeks after surgery)

Comparison: Mouth-opening exercises initiated in postoperative week 2 vs the same programme initiated in week 4. Tool: RoB 2 for individually randomized parallel-group trials.

Domain	Judgement	Supporting reason
D1. Randomization process	Some concerns	An independent statistician generated a computer-based random sequence and opaque sealed envelopes were used for allocation concealment. However, the report is internally inconsistent regarding whether 72 or 76 participants were randomized; no important baseline imbalance was observed.
D1b. Identification/recruitment in cluster trials	Not applicable	Individually randomized trial.
D2. Deviations from intended interventions	Low risk	Participants and intervention providers could not be blinded, but no trial-induced deviations from the assigned timing strategies were reported.
D3. Missing outcome data	Low risk	Seventy-two of 76 randomized participants completed follow-up (36/36); losses were few, balanced between groups, and unlikely to materially affect the result.
D4. Measurement of the outcome	Low risk	Maximum interincisal opening was measured directly by trained outcome assessors who were blinded to group assignment.

Domain	Judgement	Supporting reason
D5. Selection of the reported result	Some concerns	Maximum interincisal opening was identified as the primary outcome and assessment times were reported, but no sufficiently detailed prospectively available analysis plan was identified to rule out selection among eligible analyses or time points.
Overall risk of bias	Some concerns	Some concerns arising from the randomization-process reporting inconsistency and selection of the reported result.

10. Wang et al., 2019: Maximum interincisal opening (3 months after discharge)

Comparison: Mouth-opening programme plus structured telephone support vs the same programme without structured telephone support. Tool: RoB 2 for individually randomized parallel-group trials.

Domain	Judgement	Supporting reason
D1. Randomization process	Low risk	An independent researcher generated the random allocation sequence; sequentially numbered opaque sealed envelopes were opened only after baseline assessment, and groups were comparable at baseline.
D1b. Identification/recruitment in cluster trials	Not applicable	Individually randomized trial.
D2. Deviations from intended interventions	Low risk	No trial-induced deviations likely to bias the effect of assignment were reported, and analysed participants remained in their originally assigned groups.
D3. Missing outcome data	Some concerns	Sixty of 68 randomized participants were analysed (30/30). Post-randomization exclusions and losses were balanced numerically but included surgical events and missed follow-up, and no sensitivity analysis addressed the potential impact of missing outcomes.
D4. Measurement of the outcome	Low risk	Maximum interincisal opening was measured by a blinded outcome assessor using a direct objective measurement.
D5. Selection of the reported result	Some concerns	Maximum interincisal opening was identified as the primary outcome and the 3-month assessment was reported, but no sufficiently detailed prospectively available analysis plan was identified to rule out selection among eligible analyses or time points.
Overall risk of bias	Some concerns	Some concerns arising from missing outcome data and selection of the reported result.

11. Govender et al., 2020: MDADI composite score (6 months after treatment)

Comparison: SIP SMART pre-treatment swallowing intervention vs usual care. Tool: RoB 2 for individually randomized parallel-group trials.

Domain	Judgement	Supporting reason
D1. Randomization process	Low risk	Patients were randomized after consent using an online computer-generated allocation system, stratified by first-line treatment with random block permutations; no important baseline imbalance attributable to randomization was identified.
D1b. Identification/recruitment in cluster trials	Not applicable	Individually randomized trial.
D2. Deviations from intended interventions	Low risk	The trial was non-blinded, but no important trial-induced deviations from the assigned intervention strategies were reported; both groups received usual post-treatment rehabilitation.
D3. Missing outcome data	Some concerns	Six-month MDADI data were available for 12 intervention and 13 usual-care participants. Missingness included deaths and non-completion, and the complete-case effect estimate was not accompanied by an analysis demonstrating robustness to missing outcomes.
D4. Measurement of the outcome	High risk	MDADI is a patient-reported swallowing-related quality-of-life measure, and participants were aware of their assigned intervention; knowledge of allocation could plausibly influence self-reported scores.
D5. Selection of the reported result	Some concerns	Candidate swallowing outcomes and follow-up times were described in a published protocol, but this feasibility trial had no definitive efficacy primary outcome and MDADI was selected as the preferred outcome for a future trial after review of the feasibility data.
Overall risk of bias	High risk	High risk arising from measurement of the patient-reported outcome, with additional concerns about missing data and selection of the reported result.

12. Zhang Y et al., 2025: EAT-10 score (3 months after particle therapy)

Comparison: Feeding management plus swallowing training vs routine radiotherapy care and health education. Tool: RoB 2 for individually randomized parallel-group trials.

Domain	Judgement	Supporting reason
D1. Randomization process	Low risk	A computer-generated random sequence was concealed in an opaque sealed envelope and allocation was performed by a researcher not otherwise involved in the trial; baseline characteristics were comparable.
D1b. Identification/recruitment in cluster trials	Not applicable	Individually randomized trial.
D2. Deviations from intended interventions	Some concerns	The report states that intention-to-treat analysis was used, but also states that poorly compliant participants were excluded, creating uncertainty about adherence-related exclusions when estimating the effect of assignment.
D3. Missing outcome data	High risk	Overall attrition was reported as 22%. Reasons included treatment-related fatigue or oral mucositis, non-adherence, refusal of follow-up, and inability to contact participants; these reasons may be related to swallowing outcomes, and no sensitivity analysis demonstrated robustness to missing data.
D4. Measurement of the outcome	High risk	EAT-10 is a patient-reported dysphagia measure. Participants could not be blinded to the intervention, so knowledge of allocation could plausibly influence self-reported scores despite blinding of data collectors and analysts.
D5. Selection of the reported result	Low risk	The trial was prospectively registered and conducted according to a previously published protocol; EAT-10 was specified as the primary outcome and the relevant follow-up times were prespecified.
Overall risk of bias	High risk	High risk arising from missing outcome data and measurement of the patient-reported outcome.

13. Shanmugam and Pravenkumar, 2024: Dysphagia Severity Scale (6 months after surgery)

Comparison: Preoperative swallowing exercises plus standard postoperative rehabilitation vs standard postoperative rehabilitation alone. Tool: RoB 2 for individually randomized parallel-group trials.

Domain	Judgement	Supporting reason
D1. Randomization process	Some concerns	A statistician generated a computer-based randomization schedule and an independent clinical research associate assigned participants, but the mechanism used to conceal the allocation sequence at enrolment was not described.
D1b. Identification/recruitment in cluster trials	Not applicable	Individually randomized trial.
D2. Deviations from intended interventions	Low risk	No important deviations from the assigned intervention strategies were reported; postoperative rehabilitation was the same in both groups and adherence to the preoperative programme was reported as reasonable.
D3. Missing outcome data	Low risk	All 60 randomized participants were assessed at 6 months, with no attrition reported.
D4. Measurement of the outcome	Some concerns	The Dysphagia Severity Scale was derived from structured bedside swallowing tests, but the report did not state whether the outcome assessor was blinded to treatment allocation.
D5. Selection of the reported result	Some concerns	DSS and the 6-month assessment were reported as planned outcome assessments, but no prospectively available trial registration or sufficiently detailed analysis plan was identified to rule out selection among eligible analyses.
Overall risk of bias	Some concerns	Some concerns arising from allocation concealment, outcome measurement, and selection of the reported result.

Abbreviations: D1, bias arising from the randomization process; D1b, bias arising from the timing of identification or recruitment of participants in cluster-randomized trials; D2, bias due to deviations from intended interventions; D3, bias due to missing outcome data; D4, bias in measurement of the outcome; D5, bias in selection of the reported result; DSS, Dysphagia Severity Scale; EAT-10, Eating Assessment Tool-10; MASA-OC, Mann Assessment of Swallowing Ability-Oral Cancer; MDADI, MD Anderson Dysphagia Inventory.
